# An Unbalanced Weighted Sequential Fusing Multi-Sensor GM-PHD Algorithm

**DOI:** 10.3390/s19020366

**Published:** 2019-01-17

**Authors:** Han Shen-Tu, Hanming Qian, Dongliang Peng, Yunfei Guo, Ji-An Luo

**Affiliations:** 1 Institution of Information and Control, Hangzhou Dianzi University, Hangzhou 310018, China; dlpeng@hdu.edu.cn (D.P.); gyf@hdu.edu.cn (Y.G.); luojian@hdu.edu.cn (J.-A.L.); 2 Science and Technology on Near-surface Detection Laboratory, Wuxi 214035, China

**Keywords:** random finite sets, multi-sensor multi-target tracking, multi-sensor data fusing, GM-PHD

## Abstract

In this paper, we study the multi-sensor multi-target tracking problem in the formulation of random finite sets. The Gaussian Mixture probability hypothesis density (GM-PHD) method is employed to formulate the sequential fusing multi-sensor GM-PHD (SFMGM-PHD) algorithm. First, the GM-PHD is applied to multiple sensors to get the posterior GM estimations in a parallel way. Second, we propose the SFMGM-PHD algorithm to fuse the multi-sensor GM estimations in a sequential way. Third, the unbalanced weighted fusing and adaptive sequence ordering methods are further proposed for two improved SFMGM-PHD algorithms. At last, we analyze the proposed algorithms in four different multi-sensor multi-target tracking scenes, and the results demonstrate the efficiency.

## 1. Introduction

The Multi-sensor Multi-target Tracking (MMT) technique generally refers to the process of estimating the targets’ number and dynamic states from multi-sensor observations. The MMT technique has many important applications in the fields of navigation, transportation, monitoring, tracking as well as remote sensing [1,2], etc. Although we can complete a multi-target tracking work by a single sensor, many factors may degrade the tracking results, such as detected missing, false alarm and observation deviation [3]. A reasonable way to overcome the weakness of single sensor tracking is to take the advantage of the redundancy of multi-sensor data. Therefore we can improve the tracking results by using MMT. It is worth mentioning that a MMT problem is usually more complex than a single sensor multi-target tracking (SMT) problem. Generally, for a MMT problem, we need to solve the multi-sensor data fusing problem besides the SMT problem.

Like most multi-target tracking problems, for a MMT problem, the MMT problem requires the mapping relationships between observations and targets. Traditional methods usually use the data association (DA) technique to discover the mapping relationship before getting point and track estimations. The DA based MMT algorithms include the Nearest Neighbor (NN) algorithm [3], the Probability Data Association (PDA) algorithm [4], the Joint Probability Data Association (JPDA) algorithm [5], the Multiple Hypotheses Tracking (MHT) algorithm [6] and the Probability Multiple Hypotheses Tracking (PMHT) algorithm [7], etc. Beside the DA based MMT algorithms, the Random Finite Set (RFS) theory provides other ways of solving the MMT problem [8,9]. For the RFS based MMT algorithms, there are two classes of methods for obtaining the trajectory estimates: (1) The multi-target state estimation (point estimation) can be achieved without a DA process in the first place. Then, one can apply a DA technique to further obtain the multi-target track estimation [10,11]. (2) One also can directly obtain trajectory estimates by using a PHD filter on the sets of trajectories [12]. Recently, the RFS technique has been studied extensively, and many applicable algorithms are proposed, such as the Probability Hypothesis Density (PHD) algorithm [13], the Cardinal Probability Hypothesis Density (CPHD) algorithm [14], the Bernoulli Tracking (BT) algorithm [15], etc.

For the DA based MMT algorithms, we often solve the SMT problem to obtain track estimations in the first place. Then, we fuse the multi-sensor track estimations [16,17,18]. Therefore, the key issue is how to solve the DA problem in the SMT and multi-sensor track fusing process. The RFS technique provides different ways to solve the MMT problem. First, one can use the RFS technique to solve the multi-sensor multi-target state estimation (point estimation) problem in the first place. Second, one can further apply the DA technique to obtain the multi-target track estimation. Moreover, the RFS technique provides flexible ways to fuse the multi-sensor data. Specifically, we can fuse the multi-sensor data at the finite set statistical estimation layer, or at the multi-sensor state estimations layer, or at the multi-sensor track estimations layer, etc. 

Mahler proposed a formalized PHD-MMT fusing framework from a theoretical perspective [19,20]. It is difficult to realize an optimal PHD-MMT algorithm for the limitation of calculation amount, thus, sub-optimal PHD-MMT algorithms are proposed [21,22,23]. The proposed PHD-MMT methods can be broadly classified into two categories, the unified fusing method and the sequential fusing method. The main feature of the unified fusing method is that it fuses the multi-sensor information in one cycle to obtain the tracking results [24,25,26]. The advantage of the unified fusing method is it can get less information loss in the fusing process, and the drawback is it may consume more computational burden. The characteristic of the sequential fusing method is that it fuses the multi-sensor information in a sequential way [27,28]. Zhang proposed a way to fuse the multi-sensor measurements in a sequential way [29] and Pao proposed a method for sequential fusing the posterior state estimations based on the JPDA algorithm [30]. The merit of the sequential fusing method is that the computational amount is linear to the sensor quantity, while it may lose more information in the fusing process. As Meyer pointed out, for there is a certain amount of information loss in each fusing cycle, the sequential fusing method is sensitive to the multi-sensor data fusing order [31]. Mahler also pointed out that changing the fusing order will produce different multi-sensor fusing algorithms [24]. Specifically, Pao proposed a method to optimize the fusing order for the multi-sensor PDA algorithm that the data from the high quality sensor should be fused later [32]. Nagappa proposed an ordering method for the multi-sensor Iterated-Corrector algorithm that the data from the low detection rate sensor should be fused first [27]. As we can see, the multi-sensor sequence fusing order will affect the tracking quality in many scenes. Therefore, adopting a suitable fusing order will help to improve the tracking quality, vice versa. Besides, in the traditional sequential fusing methods, we often calculate the multi-sensor data fusing weights in a balanced way [27]. However, the information from the later fused sensor may be over used by adopting a balanced weighted sequential fusing method.

In this paper, we first propose a sequential fusion multi-sensor Gaussian Mixture PHD (SFMGM-PHD) algorithm. Then, we propose two improved unbalanced weighted sequential fusion multi-sensor Gaussian Mixture PHD (USFMGM-PHD) algorithms. The main contributions of this paper are as follows: (1) We propose the SFMGM-PHD algorithm which can fuse the multi-sensor information sequentially at the estimated posterior Gaussian Mixture (GM) layer. (2) We adopt the optimal sub-pattern assignment (OSPA) distance to evaluate the quality of the multi-sensor posterior GM estimations. Then, the multi-sensor adaptive sequence ordering method can be derived based on the sensor quality evaluation. (3) Two improved SFMGM-PHD algorithms are proposed based on the unbalanced weighted fusing and adaptive sequence ordering methods.

The rest of this paper is organized as follows. Section 2 is the problem formulation. In Section 3, we propose the SFMGM-PHD algorithm. Two USFMGM-PHD algorithms are proposed in Section 4. The simulation results are shown in Section 5. Section 6 draws the conclusions.

## 2. Problem Formulation

It is assumed there are $N_{k}$ targets at time k, where $N_{k}$ is an unknown variable. The state vector of target i at time k is described by $x_{k,i}$. Then, the set of $N_{k}$ targets at time k is denoted by $X_{k}=\{x_{k,1},x_{k,2},\ldots ,x_{k,N_{k}}\}$. For an arbitrary target $x_{k,i}\in X_{k}$, if it exists at time k+1, then, the state transition equation can be described as follows:

(1) $$x_{k+1,i}=f_{k,k+1}(x_{k,i})+w_{k,i}$$

where, $f_{k,k+1}$ is the one-step state transition function, and $w_{k,i}$ is the un-modeled error.

It is assumed there are s sensors. For an arbitrary sensor j, its target detection rate for a particular target is $0\leq P_{d,j}\leq 1$. If at time k, target $x_{k,j}$ is observed by sensor j, then, the observation equation can be described as follows:

(2) $$z_{k,j}=g_{k,j}(x_{k,i})+v_{k,i,j}$$

where, $z_{k,j}$ is the observation vector, $g_{k,j}$ is the observation function, and $v_{k,i,j}$ is the observation error, which is assumed to be a Gaussian white noise with zero mean and covariance $R_{j}$.

Except when receiving the data from targets, the sensor also receives false alarms from clutters. At time k, the received clutter number for sensor j submits to a Poisson distribution with intensity $\lambda_{j}$. The clutters take their positions uniformly in the target space. Specifically, the clutters received by sensor j at time k are described as follows:

(3) $$\left\{ \begin{array}{l} \rho (n_{k})=(e^{-\lambda_{j}}\lambda_{j}^{n_{k}})/n_{k}!\;n_{k}=0,1,2,\ldots \\ q(z_{j,l}^{c})=1/\Psi (x)\;\;l=1,2,\ldots ,n_{k} \end{array}\right.$$

where, $n_{k}$ presents the observed clutter number, $\rho (n_{k})$ is the corresponding probability, $q(z_{j,l}^{c})$ is the probability density of clutter l taking its position at $z_{j,l}^{c}$, and Ψ(x) is the volume of the observation space.

The observations of sensor j at time k are denoted by $Z_{k}^{j}=\{z_{j,k}^{1},\ldots ,z_{j,k}^{r}\}$, the observations of sensor j until time k are presented by $Z_{1:k}^{j}=\{Z_{1}^{j},\ldots ,Z_{k}^{j}\}$, and the accumulated observations for all s sensors are summarized by $Z_{1:k}^{1:s}=\{Z_{1:k}^{1},\ldots ,Z_{1:k}^{s}\}$. In this paper, the object of MMT is to estimate the numbers and states of the unknown targets based on $Z_{1:k}^{1:s}$.

## 3. Sequential Fusion Multi-Sensor Gaussian Mixture PHD Algorithm

To solve the problem described in Section 2, we propose the Sequential fusion multi-sensor Gaussian Mixture PHD (SFMGM-PHD) algorithm in this section based on the GM-PHD algorithm [33]. To facilitate discussion, we first review the basic conclusions of the single sensor PHD algorithm. Then, the details of the SFMGM-PHD algorithm will be explained. 

### 3.1. A Briefl Review for the Single-Sensor PHD Algorithm

For sensor j, if the target motion and observation models are described by Equations (1)–(3), then, the formalized single sensor PHD algorithm can be described by Equations (4)–(6) [13,14]:

(4) $$D_{k|k-1}(x_{k}|Z_{1:k-1}^{j})=\int f_{k|k-1}(x|\tau )D_{k-1|k-1}(\tau )d\tau$$

(5) $$D_{k|k}(x|Z_{1:k}^{j})=(1-P_{d,j})D_{k|k-1}(x|Z_{1:k-1}^{j})+\sum_{z\in Z_{k}^{j}}\frac{P_{d,j}g_{k}(z|x)D_{k|k-1}(x|Z_{1:k-1}^{j})}{\kappa_{k}(z)+\int P_{d,j}g_{k}(z|x)D_{k|k-1}(x|Z_{1:k-1}^{j})dx}$$

(6) $$\kappa_{k}(z)=\lambda_{j}\sum_{z\in Z_{k}^{j}}q(z)$$

where, $D_{k|k}(x|Z_{1:k}^{j})$ presents the posterior PHD estimation, $f_{k|k-1}(x|\tau )$ is the target state transition function, $P_{d,j}$ is the target detection rate, $g_{k}(z|x)$ is the observation likelihood, and $\kappa_{k}(z)$ reflects the clutter intensity.

Generally, it is difficult to obtain the closed-form solution of Equations (4) and (5) for the integral operations that are involved. Therefore, suboptimal methods are proposed, such as the Particle Filter PHD (PF-PHD) algorithm [34] and the GM-PHD algorithm [33], where the integral operation can be replaced by the summation of the particles or Gaussian Mixtures, respectively.

### 3.2. The SFMGM-PHD Algorithm

**Figure 1:**
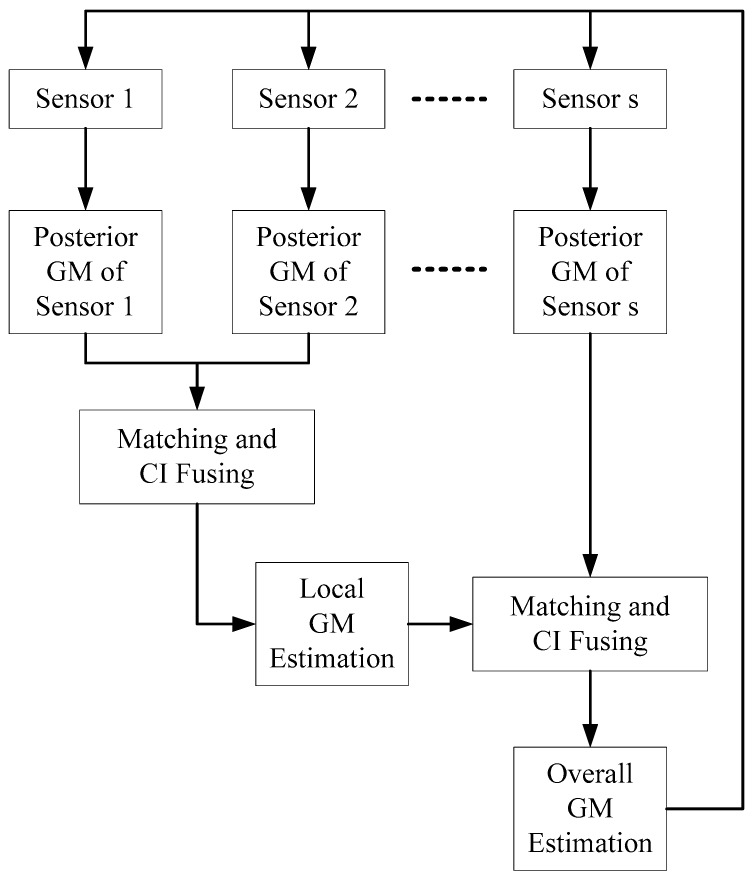
The framework of the SFMGM-PHD algorithm.

In this section, the SFMGM-PHD algorithm will be proposed through the fusion of the multi-sensor posterior GM estimations. As depicted in Figure 1, we assumed that there are s sensors. First, each sensor receives its posterior GM estimation by a single sensor GM-PHD algorithm. Secord, we fuse the posterior GM estimations of sensors 1 and 2 to possess the local posterior GM estimation, based on the matching and CI fusing [35,36] operations. Third, we fuse the local posterior GM estimations with the GM estimations of the remaining sensors sequentially, to achieve the overall posterior GM estimation. At last, the fused GM estimation will be fed back to the local sensors. The details are as follows.

**(1) Single Sensor Posterior GM Estimation**

**① Prediction:**

It is assumed that the posterior GM estimation of sensor j at time k−1 is available as ${\left\{\omega_{S,k-1|k-1}^{j,i},m_{S,k-1|k-1}^{j,i},P_{S,k-1|k-1}^{j,i}\right\}}_{i=1}^{J_{k-1}}$. If the target survival probability is $q_{s}$, then, the predictive GM estimation for the survival target can be calculated by Equation (7):

(7) $$D_{S,k|k-1}^{j}\left(x\right)=q_{S}\sum_{i=1}^{J_{k-1}}\omega_{S,k|k-1}^{j,i}N\left(x;m_{S,k|k-1}^{j,i},P_{S,k|k-1}^{j,i}\right)$$

where, $\left\{\omega_{S,k-1|k-1}^{j,i},m_{S,k-1|k-1}^{j,i},P_{S,k-1|k-1}^{j,i}\right\}$ is a Gaussian Mixture with a mean $m_{S,k-1|k-1}^{j,i}$ and a covariance $P_{S,k-1|k-1}^{j,i}$. 

At time k, the newborn GM estimation for sensor j is described by Equation (8):

(8) $$\gamma_{k}^{j}\left(x\right)=\sum_{i=1}^{J_{\gamma ,k}}\omega_{\gamma ,k}^{j,i}N\left(x;m_{\gamma ,k}^{j,i},P_{\gamma ,k}^{j,i}\right)$$

Then, the total predictive GM estimation for sensor j at time k is calculated by the following equation:

(9) $$D_{k/k-1}^{j}\left(x\right)=D_{S,k|k-1}^{j}\left(x\right)+\gamma_{k}^{j}\left(x\right)$$

Here, the predictive GM number is $J_{k/k-1}=J_{k-1}+J_{\gamma ,k}$, $J_{k-1}$ is the successive part, and $J_{\gamma ,k}$ is the newborn part.

**② Update:**

If the observation of sensor j at time k is available as $Z_{k}^{j}=\{z_{j,k}^{1},\ldots ,z_{j,k}^{r}\}$, then we can use Equation (10) to obtain the posterior GM estimation:

(10) $$D_{k}^{j}\left(x\right)=\left(1-P_{d}^{j}\right)D_{k|k-1}^{j}\left(x\right)+\sum_{z_{j,k}^{l}\in Z_{k}^{j}}\sum_{i=1}^{J_{k|k-1}}\omega_{k}^{i}(z_{j,k}^{l})N\left(x;m_{k|k}^{i},P_{k|k}^{i}\right)$$

where, $\omega_{k}^{i}(z_{j,k}^{l})$ can be calculated as follows:

(11) $$\omega_{k}^{i}(z_{j,k}^{l})=\frac{P_{d,j}\cdot \omega_{k/k-1}^{i}q_{k}^{i}(z_{j,k}^{l})}{\kappa_{k}(z_{j,k}^{l})+P_{d}^{j}\sum_{m=1}^{J_{k/k-1}}\omega_{k/k-1}^{m}q_{k}^{m}(z_{j,k}^{l})}$$

where, $q_{k}^{i}(z_{j,k}^{l})$ and $\kappa_{k}(z_{j,k}^{l})$ are the likelihood functions of target and clutter, respectively.

Then, for sensor j=1,2,…,s, we have the posterior GM estimations as ${\left\{\omega_{k}^{j,i},m_{k}^{j,i},P_{k}^{j,i}\right\}}_{i=1}^{J_{k}^{j}}$, where $J_{k}^{j}=(1+r)\cdot J_{k|k-1}$, for the unobserved part, $\omega^{j,i}=(1-P_{d}^{j})\cdot \omega_{k|k-1}^{i}$, for the observed part, $\omega^{j,i}=\omega_{k}^{i}(z_{j,k}^{l})$, and $m_{k}^{j,i}$ and $P_{k}^{j,i}$ can be achieved by an embedded Kalman Filter [33].

**(2) Multi-sensor GM Estimation Matching and Fusing**

If the posterior GM estimations of sensor j=1,2,…,s are available at time k, then, we can construct the multi-sensor GM estimation sequential fusing algorithm according to the fusing framework described by Figure 1; the details are listed in Algorithm 1.

**Algorithm 1** The multi-sensor Gaussian Mixture (GM) estimation sequential fusing algorithmFor p=1,2,…,s−1 do (*) **Step 1:** If p=1, let ${\left\{{\bar{\omega }}_{k}^{p,i},{\bar{m}}_{k}^{p,i},{\bar{P}}_{k}^{p,i}\right\}}_{i=1}^{{\bar{J}}_{k}^{p}}={\left\{\omega_{k}^{1,i},m_{k}^{1,i},P_{k}^{1,i}\right\}}_{i=1}^{J_{k}^{1}}$ be the current local GM estimation. If p>1, inherit the local GM estimation as ${\left\{{\bar{\omega }}_{k}^{p,i},{\bar{m}}_{k}^{p,i},{\bar{P}}_{k}^{p,i}\right\}}_{i=1}^{{\bar{J}}_{k}^{p}}={\left\{{\bar{\omega }}_{k}^{p-1,i},{\bar{m}}_{k}^{p-1,i},{\bar{P}}_{k}^{p-1,i}\right\}}_{i=1}^{{\bar{J}}_{k}^{p-1}}$. **Step 2:** We obtain the posterior GM estimations of sensor p+1 as ${\left\{\omega_{k}^{p+1,i},m_{k}^{p+1,i},P_{k}^{p+1,i}\right\}}_{i=1}^{J_{k}^{p+1}}$. For $i=1,2,\ldots ,J_{k}^{p+1}$ do (**)  For $j=1,2,\ldots ,{\bar{J}}_{k}^{p}$ do (***)  Compute the Euclidean distance between $\left\{\omega_{k}^{p+1,i},m_{k}^{p+1,i},P_{k}^{p+1,i}\right\}$ and $\left\{{\bar{\omega }}_{k}^{p,j},{\bar{m}}_{k}^{p,j},{\bar{P}}_{k}^{p,j}\right\}$, denoted by L(i,j), where $i\in \{1,2,\ldots ,J_{k}^{p+1}\}$, $j\in \{1,2,\ldots ,{\bar{J}}_{k}^{p}\}$.  End (***)  ① Find the GM pair $\left\{\left\{\omega_{k}^{p+1,i},m_{k}^{p+1,i},P_{k}^{p+1,i}\right\},\left\{{\bar{\omega }}_{k}^{p,j},{\bar{m}}_{k}^{p,j},{\bar{P}}_{k}^{p,j}\right\}\right\}$ with the minimum Euclidean distance $L{\left(i,j\right)}_{\min}$.  ② Set a threshold D. If $L{\left(i,j\right)}_{\min}>D$, then, delete GM $\left\{\omega_{k}^{p+1,i},m_{k}^{p+1,i},P_{k}^{p+1,i}\right\}$ from the set ${\left\{\omega_{k}^{p+1,i},m_{k}^{p+1,i},P_{k}^{p+1,i}\right\}}_{i=1}^{J_{k}^{p+1}}$ and put it into the supplementary GM set $M_{\sup}$.  ③ If $L{\left(i,j\right)}_{\min}\leq D$, then use Equations (12)–(14) to fuse $\left\{\omega_{k}^{p+1,i},m_{k}^{p+1,i},P_{k}^{p+1,i}\right\}$ and $\left\{{\bar{\omega }}_{k}^{p,j},{\bar{m}}_{k}^{p,j},{\bar{P}}_{k}^{p,j}\right\}$ into $\left\{\omega_{F},m_{F},P_{F}\right\}$: (12)$$\omega_{F}=(\omega_{k}^{p+1,i}+{\bar{\omega }}_{k}^{p,j})/2$$
(13)$$\left\{ \begin{array}{l} \pi^{i}=\omega_{k}^{p+1,i}/(\omega_{k}^{p+1,i}+{\bar{\omega }}_{k}^{p,j})\\ \pi^{j}={\bar{\omega }}_{k}^{p,j}/(\omega_{k}^{p+1,i}+{\bar{\omega }}_{k}^{p,j}) \end{array}\right.$$
(14)$$\left\{ \begin{array}{l} m_{F}=P^{F}\cdot \left(\pi^{i}(P_{k}^{p+1,i}m_{k}^{p+1,i})+\pi^{j}({\bar{P}}_{k}^{p,j}{\bar{m}}_{k}^{p,j})\right)\\ P^{F}={\left(\pi^{i}{\left(P_{k}^{p+1,i}\right)}^{-1}+\pi^{j}{\left({\bar{P}}_{k}^{p,j}\right)}^{-1}\right)}^{-1} \end{array}\right.$$  Then, we put $\left\{\omega_{F},m_{F},P_{F}\right\}$ into the supplementary GM set $M_{\sup}$, $\left\{\omega_{k}^{p+1,i},m_{k}^{p+1,i},P_{k}^{p+1,i}\right\}$ and $\left\{{\bar{\omega }}_{k}^{p,j},{\bar{m}}_{k}^{p,j},{\bar{P}}_{k}^{p,j}\right\}$ are deleted from ${\left\{\omega_{k}^{p+1,i},m_{k}^{p+1,i},P_{k}^{p+1,i}\right\}}_{i=1}^{J_{k}^{p+1}}$ and ${\left\{{\bar{\omega }}_{k}^{p,i},{\bar{m}}_{k}^{p,i},{\bar{P}}_{k}^{p,i}\right\}}_{i=1}^{{\bar{J}}_{k}^{p}}$, respectively. End (**) **Step 3:** We obtain the updated local GM estimation as follows:(15)$${\left\{{\bar{\omega }}_{k}^{p,i},{\bar{m}}_{k}^{p,i},{\bar{P}}_{k}^{p,i}\right\}}_{i=1}^{{\bar{J}}_{k}^{p}}⇐{\left\{{\bar{\omega }}_{k}^{p,i},{\bar{m}}_{k}^{p,i},{\bar{P}}_{k}^{p,i}\right\}}_{i=1}^{{\bar{J}}_{k}^{p}}\cup M_{\sup}$$ End (*) **Step 4:** Output the fused posterior GM estimation.

For convenience, we further summarize the SFMGM-PHD algorithm in Algorithm 2.

**Algorithm 2** A simple list of the SFMGM-PHD algorithm**Setup 1:** Use Equations (7)–(9) to calculate the multi-sensor predictive GM estimations. **Step 2:** Use Equations (10) and (11) to obtain the multi-sensor updated GM estimations. **Step 3:** Fuse the multi-sensor posterior GM estimations by the algorithm described in Algorithm 1. The fused GM estimations are feedback to local sensors as the preliminary data in step 1. **Step 4:** Some pruning, merging, clustering, and association methods [10,28] can be applied to achieve the targets’ number and state estimations.

**Remark** **1.** *In Section 3, the SFMGM-PHD algorithm is proposed for the MMT problem by fusing the multi-sensor GM estimations in a sequential way. However, two problems are not considered by the normal SFMGM-PHD algorithm. First, the normal SFMGM-PHD algorithm does not consider the problem of the multi-sensor fusing order. As mentioned in Section 1, the sequential fusing method is sensitive to the fusing order [31], so that adopting an unsuitable fusing order may degrade the fusing quality. By observing Equations (12) and (13), we can see the second problem is that the SFMGM-PHD algorithm adopts a balanced weighted fusing method. Specifically, the SFMGM-PHD tracker calculates the fusing weights for all sensors in a balanced way, and the balances method will in fact pay more attention to the GM estimations from the later fused sensors than those from the earlier fused sensors. In normal cases, there is no need for us to pay more attention to the GM estimations from the later fused sensors, so that we need to pursuit an unbalanced weighted fusing method that can fuse the multi-sensor GM estimations in a non-discriminatory way.*

## 4. Unbalanced Weighted Sequential Fusion Multi-Sensor Gaussian Mixture PHD Algorithm

**Figure 2:**
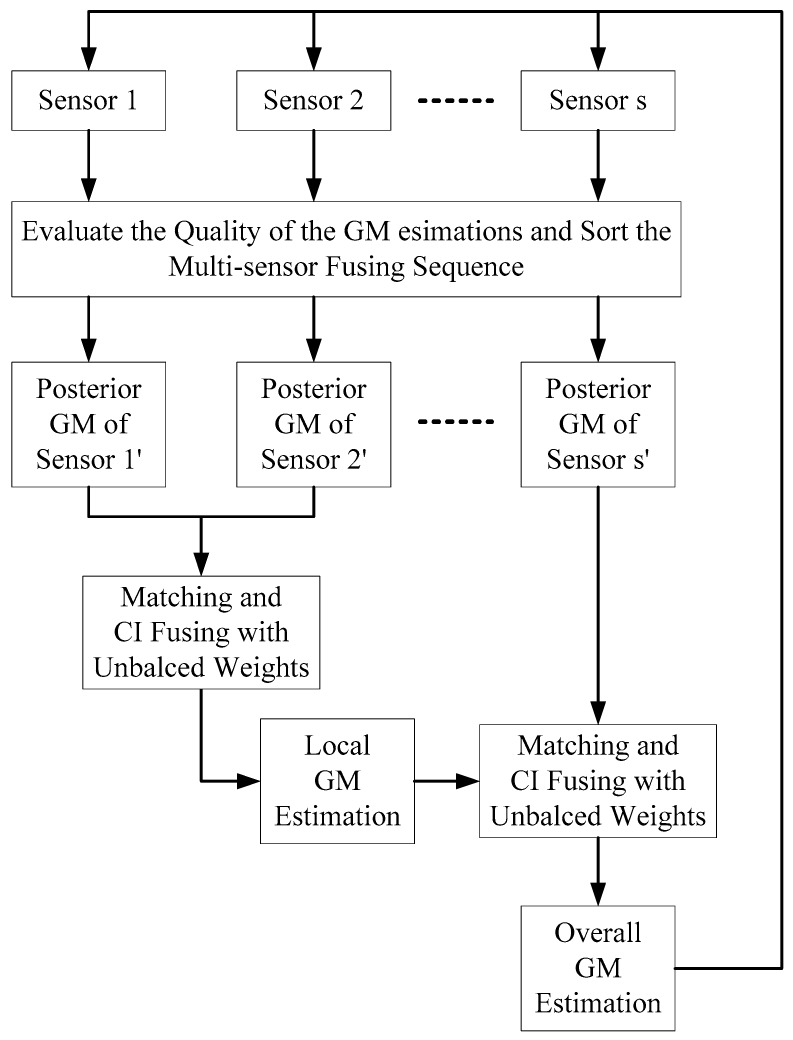
The framework of the USFMGM-PHD algorithm.

In this section, two USFMGM-PHD algorithms are proposed to solve the above-mentioned problems in Remark 1. As depicted in Figure 2, the USFMGM-PHD algorithm adopts a similar framework as described in Figure 1. The improvements are as follows. (1) First, the OSPA metric [34] is employed to value the quality of GM estimations of each sensor. Then, we can sort the multi-sensor data fusing sequence from the higher quality sensor to the lower quality sensor. (2) Second, an unbalanced weighted multi-sensor sequential fusing method is proposed, to eliminate the discrimination for the different sensors. 

### 4.1. Sorting the Multi-Sensor Fusing Sequence Based on the OSPA Metric

It is assumed that the posterior GM estimations of all s sensors at time k are available as ${\left\{\omega_{k}^{j,i},m_{k}^{j,i},P_{k}^{j,i}\right\}}_{i=1}^{J_{k}^{j}}$, j=1,…,s. Then, for two sensors $j_{1}\neq j_{2}$, we can use the OSPA metric to measure the consistency between the two sensors, by Equation (16):

(16) $$C_{j_{1},j_{2}}^{k}={\left(\frac{1}{J_{k}^{j}}\left(\underset{l\in \{1,\ldots ,J_{k}^{j_{2}}\}}{\min}\sum_{i=1}^{J_{k}^{j_{1}}}\|m_{k}^{j_{1},i},m_{k}^{j_{2},l}{\|}^{p}+c^{p}|J_{k}^{j_{1}}-J_{k}^{j_{2}}|\right)\right)}^{1/p}$$

where, c is the blending coefficient that can create a balance between the spacing and the quantity, and 1≤p≤+∞ is the sensitive factor, which can be normally selected as 1 or 2.

**Definition** **1.** *The overall consistency value of sensor* j *at time* k *, denoted by* $C_{j_{1}}^{k}$ *, is defined by Equation (17):*

(17) $$C_{j_{1}}^{k}=\sum_{j_{2}=1,j_{1}\neq j_{2}}^{s}C_{j_{1},j_{2}}^{k}$$

Based on Definition 1, we can calculate the overall consistency values for all sensors. We assume that the majority of the sensors can provide high quality estimates, and the minority of the sensors provide inferior estimates. Therefore, we think if a sensor is consistent with the majority, there is a high probability that this sensor is a high quality one. Then, the sensor with smaller overall consistency value can provide better GM estimates. As the consequence, we sort the multi-sensor fusing sequence from small to large with respect to the overall consistency value (OCV).

### 4.2. Unbalanced Weighted Multi-Sensor Sequential Fusing Method

Obviously, the sequential fusing time slots for the s sensors is s−1. When processing the n-th fusion, 1≤n≤s−1, we need to fuse the GM estimations from the (n+1)-th sensor with the previously fused GM estimations. In order to derive non-discriminatory fusing weights, we reform Equation (13) in an unbalanced way, as Equation (18):

(18) $$\left\{ \begin{array}{l} \pi^{i}=\frac{{\bar{\omega }}_{k}^{p,j}}{\left(\omega_{k}^{p+1,i}+{\bar{\omega }}_{k}^{p,j})\cdot (n+1\right)}\\ \pi^{j}=1-\pi^{i} \end{array}\right.$$

### 4.3. The Pseudo-Codes of Two USFMGM-PHD Algorithms

In this section, we summarize two USFMGM-PHD algorithms in Algorithms 3 and 4. We name them as USFMGM-PHDA and USFMGM-PHDB respectively. The two USFMGM-PHD algorithms have the similar frameworks. The difference is that USFMGM-PHDB adopts the adaptive sorting and unbalanced weighted fusing methods that is proposed in Section 4.1 and Section 4.2, whereas USFMGM-PHDA only adopts the unbalanced weighted fusing method that is proposed in Section 4.2.

**Algorithm 3** A simple list of the USFMGM-PHDA algorithms.**Step 1:** Use Equations (7)–(9) to calculate the multi-sensor predictive GM estimations. **Step 2:** Use Equations (10) and (11) to obtain the multi-sensor updated GM estimations. **Step 3** Fuse the multi-sensor posterior GM estimations by the algorithm described in Algorithm 1, where Equation (13) is replaced by Equation (18) (see Section 4.2). The fused GM estimations are fed back to local sensors as the preliminary data in Step 1. **Step 4:** Some pruning, merging, clustering, and association methods [10,28] can be applied here to achieve the targets’ number and state estimations. 

**Algorithm 4** A simple list of the USFMGM-PHDB algorithm.**Step 1:** Use Equations (7)–(9) to calculate the multi-sensor predictive GM estimations. **Step 2:** Use Equations (10) and (11) to obtain the multi-sensor-updated GM estimations. **Step 3:** Before fusing the multi-sensor posterior GM estimations, use Equations (16) and (17) to sort the multi-sensor fusing sequence from small to large with respect to the overall consistency value (see Section 4.1). **Step 4:** Fuse the multi-sensor posterior GM estimations by the algorithm described in Algorithm 1, where Equation (13) is replaced by Equation (18) (see Section 4.2). The fused GM estimations are fed back to the local sensors as the preliminary data in step 1. **Step 5:** Some pruning, merging, clustering, and association methods [10,28] can be applied to achieve the targets’ number and state estimations.

## 5. Simulations

In the simulations, four multi-sensor multi-target tracking scenes are considered with different detection and false alarm settings. Three proposed multiple-sensor GM-PHD algorithms will be analyzed. The first algorithm is the SFMGM-PHD proposed in Section 3, the second and third algorithms are the USFMGM-PHDA and USFMGM-PHDB proposed in Section 4. The SGM-PHD algorithm [28] and the sequential measurement fusion multi-sensor GM-PHD (SMFMGM-PHD) algorithm [29] will also be tested, and their results will be adopted as the benchmark. All results are achieved by 1000 Monte Carlo runs.

We set three targets in the simulations and the nearly constant velocity model was used to describe the motions:

(19) $$x_{k+1}^{i}=F\cdot x_{k}^{i}+\omega_{k}^{i}\;i=1,2,3$$

where $x_{k}^{i}=[\begin{array}{cccc} x_{k} & {\dot{x}}_{k} & y_{k} & {\dot{y}}_{k} \end{array}{]}^{T}$ presents the state vector of target i at time k, and the components are the position and velocity along the x and y coordinates, respectively. F is the state transition matrix, and $\omega_{k}^{i}$ is the unmolded noise. We set the sampling period T=1 second in the tests.

(20) $$F=\left[\begin{array}{cccc} 1 & T & 0 & 0\\ 0 & 1 & 0 & 0\\ 0 & 0 & 1 & T\\ 0 & 0 & 0 & 1 \end{array}\right]$$

We set four sensors in the simulations, and they were independent to each other. The target-derived observation was modeled by the following equation:

(21) $$z_{k}^{j}=H\cdot x_{k}+v_{k}^{j}\;j=1,2,3,4$$

where $z_{k}^{j}$ is the observation achieved by sensor j at time k. H is the observation matrix, and $v_{k}^{j}$ is the observation error, which is Gaussian and white. For i≠j, $v_{k}^{i}$ is independent to $v_{k}^{j}$. The four sensors have the same observation error covariance matrixes R.

(22) $$H=\left[\begin{array}{cccc} 1 & 0 & 0 & 0\\ 0 & 0 & 1 & 0 \end{array}\right]$$

(23) $$R=\left[\begin{array}{cc} 400 & 0\\ 0 & 400 \end{array}\right]$$

The clutter-derived observation is described by Equation (3) in Section 2. In the simulations, we set the tracking space as a [−1000,1000]×[−1000,1000] plane. Therefore, the space volume was $\Psi (x)=4\times 10^{6}$ here. The clutter intensity $\lambda_{j}$ for each sensor depended on the specific scene, and this will be explained in the following part. 

We set four tracking scenes with different detection rates and clutter intensities settings, as described in Table 1. Here, we used $P_{d}^{i}$ and $\lambda_{i}$ to present the detection rate and clutter intensity of sensor i, respectively. We set the same detection rate and clutter intensity for all sensors in scene one. In scene two, we had different detection rates for the four sensors. Specifically, the first sensor had the highest detection rate as $P_{d}^{1}=0.9$, and the fourth sensor had the lowest detection rate as $P_{d}^{1}=0.6$. In scene three, the detection rate was same for all sensors, while the clutter intensities were different. Specifically, sensor one had the weakest clutter intensity as $\lambda_{1}=20$, and sensor four had the strongest clutter intensity as $\lambda_{4}=80$. In scene four, the detection rates and clutter intensities were different for the four sensors, where sensor one had the highest detection rate and the weakest clutter intensity, and sensor four had the lowest detection rate and the strongest clutter intensity. Therefore, it can be roughly considered that the four sensors had the same quality in scene one. In the other three scenes, we had an order with respect to the sensor quality (from good to bad), where “sensor one → sensor two → sensor three → sensor four”. 

**Figure 3:**
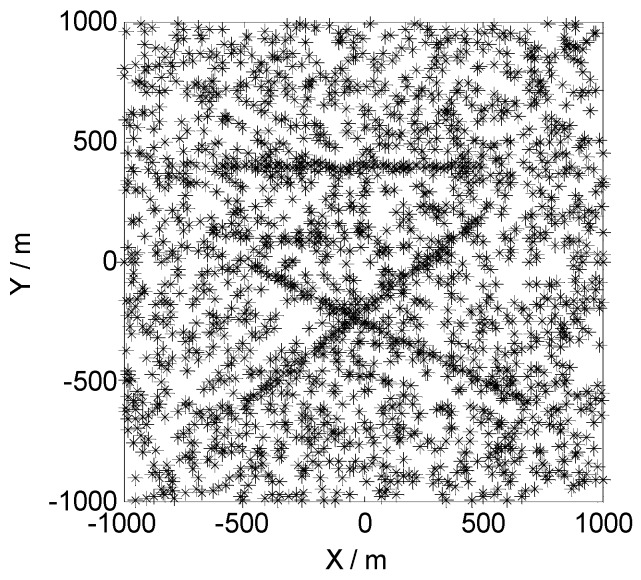
Observations of sensor one in a one-time Monte Carlo run in the first scene.

Figure 3 presents the observations of sensor one in a one-time Monte Carlo run in the first scene. As we can see, three targets appeared in the two-dimensional monitoring area. The first and second targets started at time 1 (with initial states, $x_{1}^{1}={\left[-500,10/s,-600,10/s\right]}^{T}$ and $x_{1}^{2}={\left[-600,10/s,400,0/s\right]}^{T}$) and ended at time 100. The third target started at time 20 (with initial states, $x_{1}^{3}={\left[700,-10/s,-600,10/s\right]}^{T}$) and ended at time 100. The other three sensors received similar observations, as shown in Figure 3, as the four sensors in scene one had the same detection rate and clutter intensity. In the other three scenes, the targets’ motions were the same as in scene one, while the detection rates and clutter intensities were different. The details are shown in Table 1.

**Figure 4:**
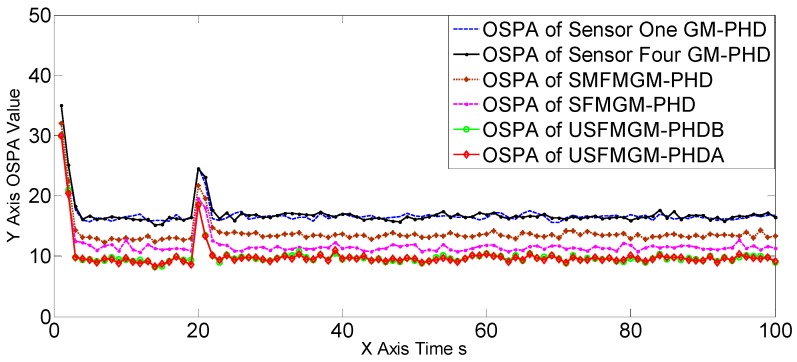
Optimal sub-pattern assignment (OSPA) estimations of the six algorithms in scene one.

**Figure 5:**
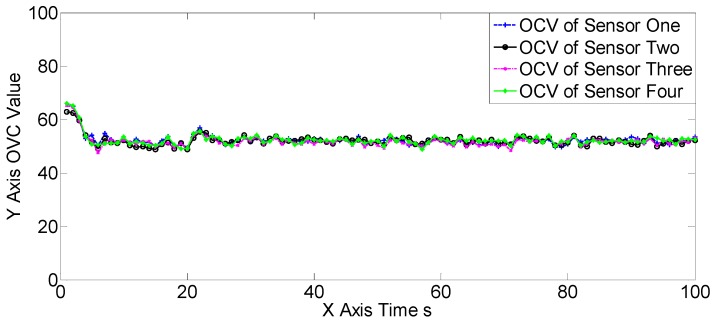
The estimated overall consistency values (OCVs) of the four sensors of the USFMGM-PHDB algorithm in scene one.

**Figure 6:**
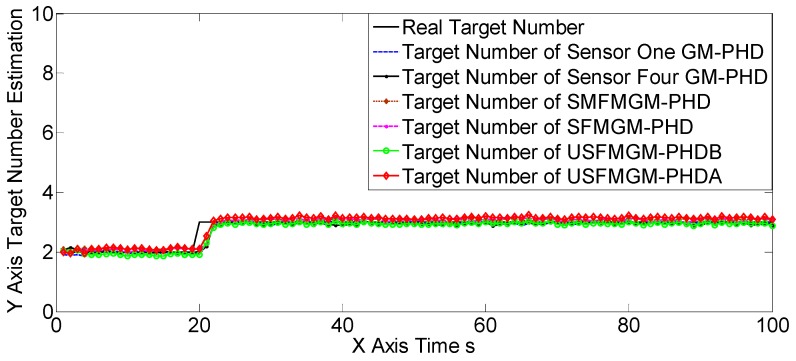
Target number estimations of six algorithms in scene one.

The OSPAs, OCVs, and the target number estimations of scene one are shown in Figure 4, Figure 5 and Figure 6, and Table 2 and Table 3. The target detection rates and clutter intensities were the same for the four sensors in this scene, thus the OSPA and target number estimation results for the two SGM-PHD algorithms were very similar. The proposed algorithms achieved obvious improvements over the SGM-PHD and SMFMGM-PHD algorithms. Besides, we noted that the USFMGM-PHDA and USFMGM-PHDB algorithms outperformed the SFMGM-PHD algorithm, as the first two algorithms adopt the unbalanced weighted fusing method (see Section 4.2). As the data qualities of four sensors were similar in scene one, the estimated OCVs of the four sensors were identical, and the fusing sequences with different orders led to similar results. Therefore, although the USFMGM-PHDB has the mechanism to sort the multi-sensor fusing sequence (see Section 4.1), the results of USFMGM-PHDA and USFMGM-PHDB are quite similar.

**Figure 7:**
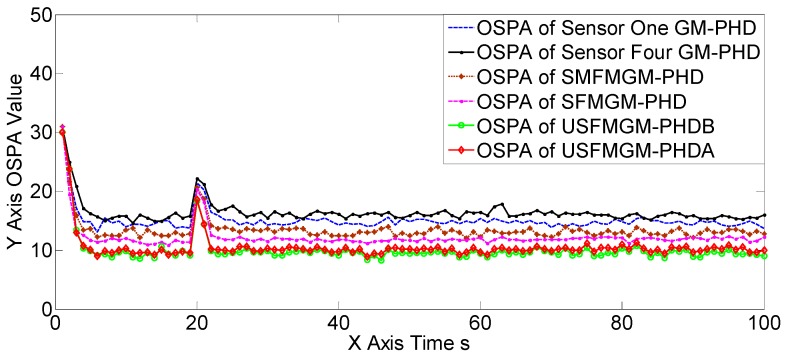
OSPA estimations of six algorithms in scene two.

**Figure 8:**
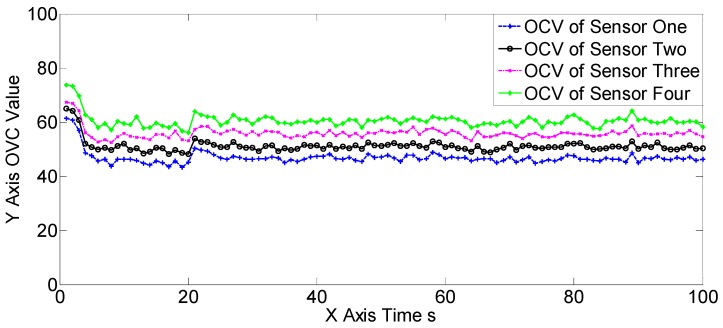
The estimated OCV of the four sensors of the USFMGM-PHDB algorithm in scene two.

**Figure 9:**
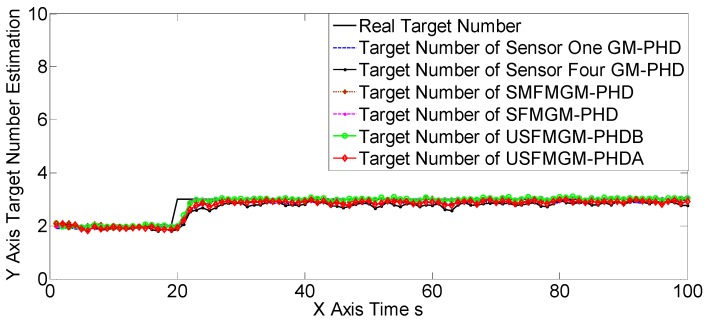
The target number estimations of six algorithms in scene two.

**Figure 10:**
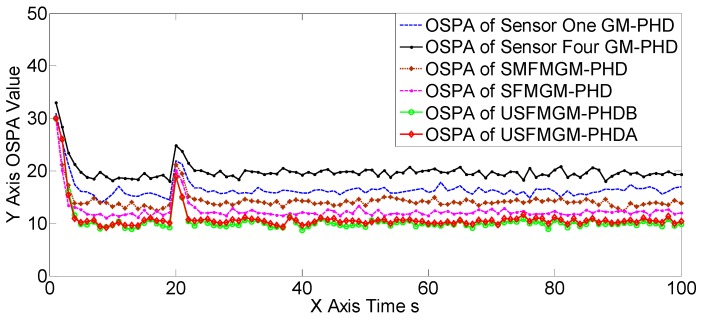
OSPA estimations of the six algorithms in scene three.

**Figure 11:**
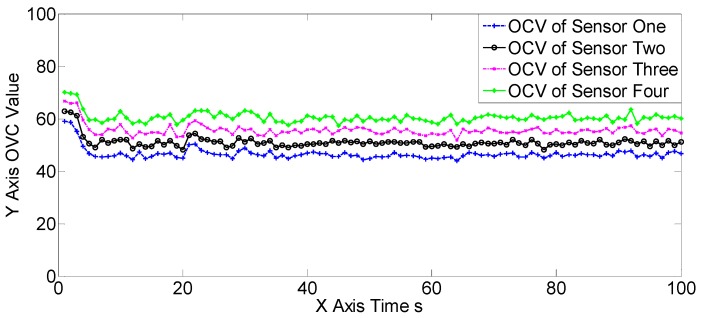
The estimated OCVs of the four sensors of the USFMGM-PHDB algorithm in scene three.

**Figure 12:**
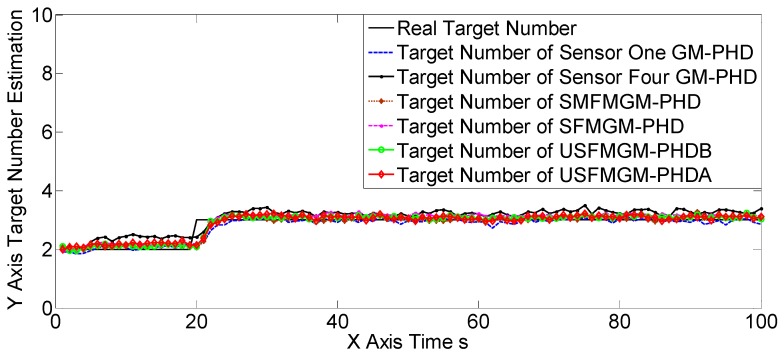
Target number estimations of the six algorithms in scene three.

**Figure 13:**
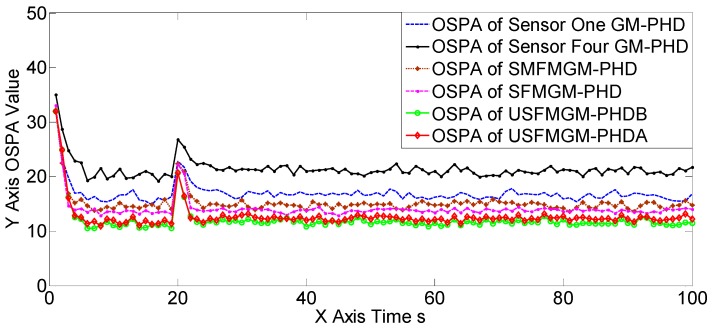
OSPA estimations of six algorithms in scene four.

**Figure 14:**
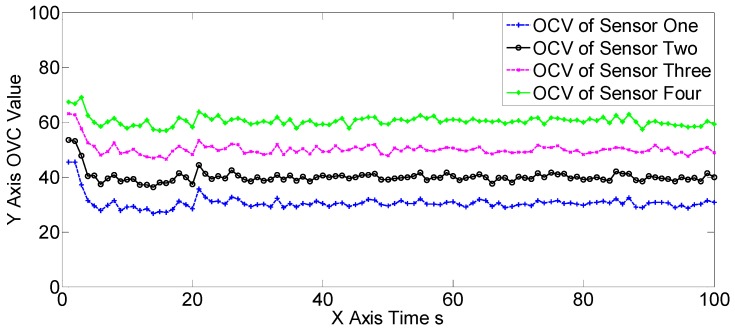
The estimated OCVs of the four sensors of the USFMGM-PHDB algorithm in scene four.

**Figure 15:**
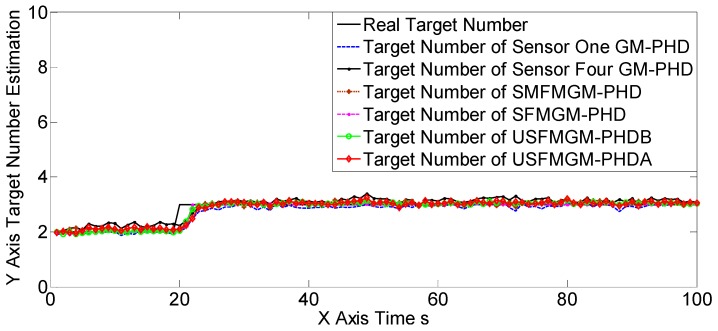
Target number estimations of the six algorithms in scene four.

The OSPA, OCV, and the target number estimations of scene two are shown in Figure 7, Figure 8 and Figure 9, Table 4 and Table 5. The OSPA, OCV, and target number estimations of scene three are shown in Figure 10, Figure 11 and Figure 12, and Table 6 and Table 7. The OSPA, OCV, and the target number estimations of scene four are shown in Figure 13, Figure 14 and Figure 15, and Table 8 and Table 9. In the above three scenes, we set different observation capacities for the four sensors. Specifically, we set the same clutter intensities and different detection rates in scene two. In scene three, we set the same detection rate and different clutter intensities. Also, in the last scene, both the detection rates and clutter intensities were different. For the sake of convenience, we set sensor one as the best sensor, and sensor four as the worst one in the above three scenes. As shown in Figure 7, Figure 8, Figure 9, Figure 10, Figure 11, Figure 12, Figure 13, Figure 14 and Figure 15 and Table 4, Table 5, Table 6, Table 7, Table 8 and Table 9, the three proposed algorithms outperformed the best SGM-PHD algorithm (sensor one) and the SMFMGM-PHD in both the OSPA and target number estimations. Among the three proposed algorithms, the USFMGM-PHDB was the best one, and the worst one was SFMGM-PHD. From Figure 8, Figure 11 and Figure 14, we see the estimated OCV reflected the sensor quality accurately; therefore the USFMGM-PHDB could obtain an optimized fusing order, based on the estimated OCV, and provide the best tracking results. Among the above three scenes, the most challenging one was scene four, and the easiest one was scene three (shown in Table 4, Table 6 and Table 8). Table 6 and Table 8 further show that the OSPA deteriorative degree of the USFMGM-PHDB algorithm was smaller than the deteriorative degree of the SFMGM-PHD algorithm. Specifically, the average OSPAs of the SFMGM-PHD algorithm in scenes three and four were 11.9580 and 14.1943, with an increase of OSPA at 15.75%; the average OSPAs of the USFMGM-PHDB algorithm in scenes three and four were 10.2914 and 12.0674, with the increase of the OSPA at 14.71%. Comparing Table 7 with Table 9, we can see the target number estimation results of the USFMGM-PHDB algorithm in scene four were even better than the results in scene three. Specifically, the average TNE deviation of the SFMGM-PHD algorithm in scene three and four are 0.0931 and 0.0992 (Table 7 and Table 9), with an increase of 6.55%. The average TNE deviations of the USFMGM-PHDB algorithm in scenes three and four were 0.0576 and 0.0527 (Table 7 and Table 9), with a decrease of 8.50%. In addition, the average OSPA of the USFMGM-PHDA algorithm in scenes two to four was 11.4098. The average OSPA of the USFMGM-PHDB algorithm in scene two to four was 10.8954. As we can see, in the last three scenes, the USFMGM-PHDB algorithm obtained an average OSPA decrease, and the USFMGM-PHDA algorithm was 4.51%. The average TNE deviations of the USFMGM-PHDA and USFMGM-PHDB algorithms were 0.0827 and 0.0594, respectively. Also, the USFMGM-PHDB algorithm obtained a TNE that was lower than the USFMGM-PHDA algorithm, at 28.17%. In general, the USFMGM-PHDA&B algorithms are better than the SFMGM-PHD algorithm, as the unbalanced weighted fusing method is adopted by the former two algorithms. Besides, the USFMGM-PHDB algorithm sorts the multi-sensor fusing sequences from the best sensor to the worst sensor, based on the OSPA consistency metric. Then, the fully utilized information will come from the best sensor rather than the worst one for the USFMGM-PHDB algorithm. As a consequence, USFMGM-PHDB shows better results than USFMGM-PHDA when the multiple sensors’ qualities are different.

We give the complexity analysis for the SFMGM-PHD, USFMGM-PHDA and USFMGM-PHDB algorithms. It is assumed that there are s sensors. At a particular tracking time stamp, the average Gaussian Mixture number of one sensor is assumed to be J, and the average observation number of one sensor is assumed to be r. We assume that the average estimated target number of one sensor at a particular tracking time stamp is n. Then, the computing complexities of the above-mentioned algorithms are shown in Table 10. As we can see, the SFMGM-PHD and USFMGM-PHDA algorithms have the same computing complexity, and the complexity of the USFMGM-PHDB algorithm is a bit larger than the other two. 

## 6. Conclusions

Three sequential fusing multi-sensor GM-PHD algorithms (SFMGM-PHD, USFMGM-PHDA&B) are proposed. First, we propose a multi-sensor posterior GM estimation sequential fusing framework by constructing the SFMGM-PHD algorithm. Then, we analyze the deficiencies of the standard SFMGM-PHD algorithm. (1) The multi-sensor GM estimations are fused with the balanced way that the information of the later fused sensor will be overused. (2) The problem of fusion sequence sorting is not considered and the fused results may be affected by the fusing sequence order. Therefore, the methods of unbalanced weighted fusing and fusion sequence sorting are further proposed to derive the USFMGM-PHDA&B algorithms. The simulation results show that the proposed algorithms can achieve better tracking results than the SGM-PHD algorithm of the best sensor and the SMFMGM-PHD algorithm in the testing scenes. Besides, the two USFMGM-PHD algorithms show better results than the SFMGM-PHD algorithm, and the best one is the USFMGM-PHDB algorithm.

There are two potential directions of future work. The first one is that we may calculate the unbalanced multi-sensor fusing weight by other ways. The second one is to find other metrics to sort the multi-sensor fusing sequence.

## Figures and Tables

**Table 1:** Four tracing scenes with different detection rates and clutter intensity settings.

	Detection Rate	Clutter Intensity
Scene one	$P_{d}^{1}=P_{d}^{2}=P_{d}^{3}=P_{d}^{4}=0.8$	$\lambda_{1}=\lambda_{2}=\lambda_{3}=\lambda_{4}=20$
Scene two	$P_{d}^{1}=0.9,P_{d}^{2}=0.8,P_{d}^{3}=0.7,P_{d}^{4}=0.6$	$\lambda_{1}=\lambda_{2}=\lambda_{3}=\lambda_{4}=20$
Scene three	$P_{d}^{1}=P_{d}^{2}=P_{d}^{3}=P_{d}^{4}=0.8$	$\lambda_{1}=20,\lambda_{2}=40,\lambda_{3}=60,\lambda_{4}=80$
Scene four	$P_{d}^{1}=0.9,P_{d}^{2}=0.8,P_{d}^{3}=0.7,P_{d}^{4}=0.6$	$\lambda_{1}=20,\lambda_{2}=40,\lambda_{3}=60,\lambda_{4}=80$

**Table 2:** OSPA estimations for scene one.

	Average OSPA	Maximum OSPA	Minimum OSPA
Sensor 1 GM-PHD	16.9157	22.0257	15.6379
Sensor 4 GM-PHD	16.9812	23.0945	15.1919
SMFMGM-PHD	13.8430	19.5509	12.2519
SFMGM-PHD	11.8608	18.1034	10.6739
USFMGM-PHDA	10.0465	13.4591	8.1692
USFMGM-PHDB	10.0089	13.3113	8.3244

**Table 3:** Target number estimations (TNE) for scene one.

	Average TNE Deviation	Maximum TNE Deviation	Minimum TNE Deviation
Sensor 1 GM-PHD	0.0932	1.0862	0.0060
Sensor 4 GM-PHD	0.1482	1.0782	0.0030
SMFMGM-PHD	0.1015	0.9953	0.0006
SFMGM-PHD	0.1265	0.9086	0.0002
USFMGM-PHDA	0.0600	0.9042	0.0002
USFMGM-PHDB	0.0561	0.8925	0.0001

**Table 4:** OSPA estimations for scene two.

	Average OSPA	Maximum OSPA	Minimum OSPA
Sensor 1 GM-PHD	14.2068	19.5765	12.6721
Sensor 4 GM-PHD	16.7701	20.6878	14.4305
SMFMGM-PHD	13.4619	18.7315	12.0467
SFMGM-PHD	12.1959	18.7781	10.9602
USFMGM-PHDA	10.7964	14.4291	9.0574
USFMGM-PHDB	10.3275	14.0350	8.2388

**Table 5:** Target number estimations (TNE) for scene two.

	Average TNE Deviation	Maximum TNE Deviation	Minimum TNE Deviation
Sensor 1 GM-PHD	0.1198	1.0763	0.0036
Sensor 4 GM-PHD	0.1050	1.0966	0.0176
SMFMGM-PHD	0.1009	1.0806	0.0054
SFMGM-PHD	0.0987	1.0260	0.0036
USFMGM-PHDA	0.0782	0.9528	0.0001
USFMGM-PHDB	0.0680	0.8981	0.0001

**Table 6:** OSPA estimations for scene three.

	Average OSPA	Maximum OSPA	Minimum OSPA
Sensor 1 GM-PHD	16.5143	21.3339	13.8544
Sensor 4 GM-PHD	19.8978	23.7430	18.0603
SMFMGM-PHD	14.3525	19.3979	12.3688
SFMGM-PHD	11.9580	17.9834	10.5322
USFMGM-PHDA	10.7097	14.7481	8.6639
USFMGM-PHDB	10.2914	14.6190	8.3410

**Table 7:** Target number estimations (TNE) for scene three.

	Average TNE Deviation	Maximum TNE Deviation	Minimum TNE Deviation
Sensor 1 GM-PHD	0.1369	0.8296	0.0035
Sensor 4 GM-PHD	0.2268	0.6772	0.0442
SMFMGM-PHD	0.1259	0.9100	0.0066
SFMGM-PHD	0.0931	1.0358	0.0001
USFMGM-PHDA	0.0841	1.1180	0.0012
USFMGM-PHDB	0.0576	0.8569	0.0001

**Table 8:** OSPA estimations for scene four.

	Average OSPA	Maximum OSPA	Minimum OSPA
Sensor 1 GM-PHD	16.9325	21.7138	14.8246
Sensor 4 GM-PHD	21.3848	25.3774	19.2107
SMFMGM-PHD	15.1855	20.8436	13.5893
SFMGM-PHD	14.1943	20.5406	12.8295
USFMGM-PHDA	12.7250	16.4566	10.9790
USFMGM-PHDB	12.0674	16.1862	10.4984

**Table 9:** Target number estimations (TNE) for scene four.

	Average TNE Deviation	Maximum TNE Deviation	Minimum TNE Deviation
Sensor 1 GM-PHD	0.1362	0.8244	0.0031
Sensor 4 GM-PHD	0.1846	1.0488	0.0205
SMFMGM-PHD	0.1189	0.9512	0.0076
SFMGM-PHD	0.0992	0.8577	0.0065
USFMGM-PHDA	0.0857	0.9438	0.0001
USFMGM-PHDB	0.0527	0.7435	0.0006

**Table 10:** The computing complexity for three algorithms.

Algorithm	SFMGM-PHD	USFMGM-PHDA	USFMGM-PHDB
Computing complexity	$O\left(sJr+sn^{2}\right)$	$O\left(sJr+sn^{2}\right)$	$O\left(sJr+(s+1)n^{2}\right)$

## References

[1] Bar-Shalom Y., Fortmann T.E. (1988). Tracking and Data Association.

[2] Martin E.L., David L.H., James L. (2009). Handbook of Multisensor Data Fusion Theory and Practice.

[3] Bar-Shalom Y. (2000). Multitarget-Multisensor Tracking: Applications and Advances.

[4] Bar-Shalom Y., Tse E. (1975). Tracking in a cluttered environment with probabilistic data association. Automation.

[5] Fortmann T., Bar-Shalom Y., Scheffe M. (1983). Sonar tracking of multiple targets using joint probabilistic data association. IEEE J. Ocean. Eng..

[6] Blackman S. (2004). Multiple hypothesis tracking for multiple target tracking. IEEE Aerosp. Electron. Syst. Mag..

[7] Cham T.J., Rehg J.M. A multiple hypothesis approach to figure tracking. Proceedings of the 1999 IEEE Computer Society Conference on Computer Vision and Pattern Recognition.

[8] Mahler R.P.S. (2007). Statistical Multisource-Multitarget Information Fusion.

[9] Mahler R. (2001). Random set theory for target tracking and identification. Multisensor Data Fusion.

[10] Kusha P., Daniel E.C., Ba-Ngu V. (2009). Data Association and Track Management for the Gaussian Mixture Probability Hypothesis Density Filter. IEEE Trans. Aerosp. Electron. Syst..

[11] Kuhsa P., Ba-Ngu V., Summetpal S. (2007). Novel Data Association Schemes for the Probability Hypothesis Density Filter. IEEE Trans. Aerosp. Electron. Syst..

[12] Angel F., Lennart S. Trajectory Probability Hypothesis Density Filter. Proceedings of the 21st International Conference on Information Fusion.

[13] Mahler R.P.S. (2003). Multitarget Bayes filtering via first-order multitarget moments. IEEE Trans. Aerosp. Electron. Syst..

[14] Vo B.T., Vo B.N., Cantoni A. (2007). Analytic implementations of the cardinalized probability hypothesis density filter. IEEE Trans. Signal Process..

[15] Papi F., Vo B.N., Vo B.T. (2015). Generalized labeled multi-Bernoulli approximation of multi-object densities. IEEE Trans. Signal Process..

[16] Toet E., Waard H.D. (1995). The Multitarget/Multisensor Tracking Problem. IEEE Trans. Signal Process..

[17] Xie Y.F., Huang Y.A., Song T.L. (2018). Iterative joint integrated probabilistic data association filter for multiple-detection multiple-target tracking. Digit. Signal Process..

[18] Yu L., Jun L., Gang L., Yao L., You H. (2017). Centralized Multi-sensor Square Root Cubature Joint Probabilistic Data Association. Sensors.

[19] Mahler R. (2009). The multisensory PHD filter, I: General solution via multitarget calculus. Proc. SPIE.

[20] Mahler R. (2009). The multisensory PHD filter, II: Erroneous solution via Poisson magic. Proc. SPIE.

[21] Nannuru S., Blouin S., Coates M., Rabbat M. (2016). Multisensor CPHD filter. IEEE Trans. Aerosp. Electron. Syst..

[22] Zhuo W.L., Shu X.C., Hao W., Ren K.H., Lin H. (2018). A Student’s Mixture Probability Hypothesis Density Filter for Multi-target Tracking with Outliers. Sensors.

[23] Saucan A.A., Coates M.J., Rabbat M. (2017). A multi-sensor multi-Bernoulli filter. IEEE Trans. Signal Process..

[24] Mahler R. Approximate multisensory CPHD and PHD filters. Proceedings of the 13th International Conference on Information Fusion.

[25] Ouyang C., Ji H. (2011). Scale unbalance problem in product multisensory PHD filter. Electron. Lett..

[26] Tian C.L., Javier P., Hong Q.F., Juan M.C. (2018). A Robust Multi-sensor PHD Filter Based on Multi-sensor Measurement Clustering. IEEE Commun. Lett..

[27] Nagappa S., Clark D.E. (2011). On the ordering of sensors in the iterated-corrector probability hypothesis density (PHD) filter. Proc. SPIE.

[28] Xu J., Huang F.M., Huang Z.L. The multi-sensor PHD filter: Analytic implementation via Gaussian mixture and effective binary partition. Proceedings of the 16th International Conference on Information Fusion.

[29] Zhang W.A., Shi L. (2018). Sequential Fusion Estimation for Clustered Sensor Networks. Automatica.

[30] Lucy Y.P., Christian W.F. A comparison of parallel sequential implementations of a multisensor multitarget tarcking algorithm. Proceedings of the 1995 American Control Conference.

[31] Meyer F. (2018). Message Passing Algorithms for Scalable Multitarget Tracking. Proc. IEEE.

[32] Lucy P., Lidia T. (2000). The Optimal Order of Processing Sensor Information in Sequential Multisensor Fusion Algorithms. IEEE Trans. Autom. Control.

[33] Ji H.Z., Mei G.G. (2018). Tracking Ground Targets with a Road Constraint Using a GMPHD Filter. Sensors.

[34] Wei J.S., Li W.W., Zhi Y.Q. (2016). Multi-Target State Extraction for the SMC-PHD Filter. Sensors.

[35] Daniel D., Thia K., Thomas L., Michael M.D. Multisensor Particle Filter Cloud Fusion for Multitarget Tracking. Proceedings of the 11th International Conference on Information Fusion.

[36] Zi L.D., Peng Z., Wen J.Q., Jin F.L., Yuan G. (2012). Sequential Covariance Intersection Fusion Kalman Filter. Inf. Sci..

